# Analysis of Factors Associated with Hypoglycemia in Patients with Out-of-Hospital Cardiac Arrest Undergoing Targeted Temperature Management

**DOI:** 10.3390/jcm14207354

**Published:** 2025-10-17

**Authors:** Wan Young Heo, Seok Jin Ryu, Dong Hun Lee, Byung Kook Lee, Yong Hun Jung, Kyung Woon Jeung

**Affiliations:** Department of Emergency Medicine, Chonnam National University Hospital, Chonnam National University of Medical School, 42, Jebong-ro, Donggu, Gwangju 61469, Republic of Korea; bean3927@naver.com (W.Y.H.); samahalak@naver.com (S.J.R.); bbukkuk@hanmail.net (B.K.L.); xnxn77@hanmail.net (Y.H.J.); neoneti@hanmail.net (K.W.J.)

**Keywords:** arrest, outcome, hypoglycemia, hypothermia

## Abstract

**Background:** Patients with out-of-hospital cardiac arrest (OHCA) are susceptible to hypoglycemia, which may worsen outcomes. Early identification of patients at risk is therefore essential. This study examined factors associated with hypoglycemia in OHCA survivors treated with targeted temperature management (TTM). **Methods:** We conducted an observational study of adults (≥18 years) with OHCA who received TTM between October 2015 and December 2024. Hypoglycemia was defined as blood glucose ≤ 70 mg/dL, assessed within 7 days of admission. The primary outcome was hypoglycemia occurrence. **Results:** Among 521 patients with OHCA, 69 (13.2%) developed hypoglycemia. Multivariable analysis identified body mass index (odds ratio [OR], 0.877; 95% confidence interval [CI], 0.808–0.953), N-terminal pro-B-type natriuretic peptide (NT-proBNP) > 2000 mg/dL (OR, 3.769; 95% CI, 2.060–6.898), and renal replacement therapy (OR, 3.429; 95% CI, 1.841–6.387) as independent factors associated with hypoglycemia. The area under the curve for the final adjusted model was 0.801 (95% CI, 0.764–0.835). **Conclusions:** In the OHCA patients who received TTM, body mass index, NT-proBNP > 2000, and renal replacement therapy were associated with hypoglycemia.

## 1. Introduction

Out-of-hospital cardiac arrest (OHCA) remains a major cause of mortality and morbidity worldwide, with the survival to hospital discharge rate being typically below 10%, despite advances in prehospital and in-hospital resuscitation efforts [1,2]. Despite significant advancements in prehospital systems of care—including increased rates of bystander cardiopulmonary resuscitation (CPR), broader implementation of public-access defibrillation, optimized emergency medical services dispatch protocols, and standardized prehospital management—recent data suggest that overall survival outcomes for OHCA remain suboptimal. A recent global meta-analysis estimated that the likelihood of achieving return of spontaneous circulation (ROSC) after an OHCA is approximately 29.7%, whereas the survival to hospital discharge is only about 8.8% [3,4]. These disparities in outcomes are largely attributable to the pathophysiological consequences of post-cardiac arrest syndrome (PCAS) that ensue following ROSC. PCAS encompasses a constellation of critical processes, including hypoxic brain injury, myocardial dysfunction, systemic ischemia–reperfusion injury, and profound metabolic dysregulation, all of which significantly influence in-hospital clinical trajectories and long-term outcomes [5]. Notably, higher cumulative doses of epinephrine administered during CPR were associated with worse clinical outcomes, including a decreased likelihood of ROSC and lower rates of favorable neurological outcome [6]. In other words, improved prehospital ROSC with higher cumulative epinephrine could bring patients with a greater ischemic burden to the hospital, but was associated with increased organ dysfunction and treatment intensity [7]. These features increase the exposure to renal replacement therapy (RRT), targeted temperature management (TTM) with tighter glycemic targets, and periods of interrupted enteral nutrition, collectively predisposing the patient to hypoglycemia during post-cardiac arrest care [8,9].

Hypoglycemia, commonly defined as a blood glucose level < 70 mg/dL, can exacerbate cerebral injury after global ischemia. Among post-arrest metabolic abnormalities, hypoglycemia has drawn increasing attention because of potential links to adverse neurological outcomes and higher mortality. In a retrospective study of in-hospital cardiac arrest, hypoglycemia was associated with in-hospital mortality in non-diabetic patients [10]. In a nationwide Japanese database, hypoglycemia (<80 mg/dL) was found to be associated with 1-month survival when compared with 80–179 mg/dL [11]. Hypoglycemia also precipitates cardiac arrhythmias and sudden death via oxidative stress, QT prolongation, and myocardial injury [12,13].

Given these consequences, early recognition of high-risk patients is critical. However, evidence on hypoglycemia risk factors in OHCA is limited. We therefore investigated hypoglycemia incidence and independent predictors of its occurrence in patients with OHCA, aiming to inform early metabolic monitoring and management in post-cardiac arrest care.

## 2. Materials and Methods

### 2.1. Study Design and Population

This prospective observational study analyzed data from adult comatose OHCA survivors treated with targeted temperature management (TTM) at Chonnam National University Hospital (Gwangju, Korea) from October 2015 to December 2024. The Institutional Review Board approved the study (CNUH-2015-164). Written informed consent was obtained from patients or legal guardians before inclusion. We included adults (≥18 years) who received TTM and excluded those in whom hemoglobin A1c (HbA1c) or N-terminal pro-B-type natriuretic peptide (NT-proBNP) could not be measured immediately after ROSC. Furthermore, patients with a mean blood glucose level greater than 250 mg/dL were excluded, consistent with a previous study [14].

### 2.2. Targeted Temperature Management

Comatose survivors underwent TTM according to guidelines. A target temperature of 33–36 °C was maintained for 24 h using either feedback-controlled endovascular catheters (Thermogard, ZOLL Medical Corporation, Chelmsford, MA, USA) or surface cooling devices (Blanketrol II; Cincinnati Sub-Zero Products, Cincinnati, OH, USA; Arctic Sun Energy Transfer Pads; Medivance, Louisville, CO, USA). After the maintenance phase, patients were rewarmed to 36.5 °C at 0.25 °C/h. All other management decisions were at the discretion of treating physicians.

### 2.3. Glucose Control During the Post-Resuscitation State

Glucose control followed a nurse-driven protocol. Based on prior studies [15], nurses maintained blood glucose at 140–180 mg/dL using intravenous insulin or glucose. Hypoglycemia was defined as ≤70 mg/dL. Glucose-containing solutions were avoided during TTM unless hypoglycemia (≤70 mg/dL) was confirmed. Blood glucose was measured hourly from arterial blood via an indwelling arterial catheter using Accu-Chek (Roche/Hitachi, Basel, Switzerland) until values stabilized within 140–180 mg/dL and then every 4 h. If hypoglycemia or severe hyperglycemia occurred, additional measurements were obtained after glucose or insulin infusion.

### 2.4. Data Collection and Primary Outcome

We extracted the following variables from hospital records: age, sex, body mass index (BMI), preexisting illness, witnessed collapse, bystander cardiopulmonary resuscitation (CPR), initial shockable rhythm, etiology of cardiac arrest, time from collapse to ROSC, serum lactate, glucose, HbA1c, NT-proBNP, partial pressure of oxygen, and partial pressure of carbon dioxide (PaCO_2_) after ROSC. We evaluated NT-proBNP > 2000 pg/mL, previously suggested as evidence of heart failure [16]. We recorded the target temperature of the TTM, any seizures, extracorporeal membrane oxygenation (ECMO), and renal replacement therapy (RRT). Within 7 days of admission, we assessed the presence of hypoglycemia and hyperglycemia > 200 mg/dL persisting despite insulin administration.

We assessed in-hospital mortality and neurological status at discharge using the Cerebral Performance Category (CPC) scale—CPC 1 (good performance), CPC 2 (moderate disability), CPC 3 (severe disability), CPC 4 (vegetative state), and CPC 5 (brain death or death) [17]. The primary outcome was hypoglycemia; the secondary outcome was a poor neurological outcome, defined as CPCs 3–5.

### 2.5. Statistical Analysis

Categorical variables are reported as counts and percentages, and continuous variables as medians with interquartile ranges because normality was not met. We compared the categorical variables using χ^2^ tests with a continuity correction for 2 × 2 tables and continuous variables using Mann–Whitney U tests.

We used multivariable logistic regression to identify factors associated with hypoglycemia. Variables with *p* < 0.05 in univariate comparisons were entered into the multivariable model. We applied a backward stepwise approach, sequentially eliminating variables with *p* > 0.10 to build the final adjusted model. The elimination process was terminated when all remaining variables had *p*-values < 0.10. BMI, NT-proBNP > 2000 pg/mL, and RRT were retained in the final adjusted model (Model 1). Logistic regression results are presented as odds ratios (ORs) with 95% confidence intervals (CIs). Kaplan–Meier survival curves were compared between the hypoglycemia and no hypoglycemia groups using the log-rank test. The prognostic performance of Model 1 for hypoglycemia was evaluated using the area under the receiver operating characteristic curve (AUROC). All analyses were performed using SPSS for Windows, version 26.0 (IBM Corp., Armonk, NY, USA) and MedCalc, version 23.0 (MedCalc Software, bvba, Ostend, Belgium). Two-sided *p* < 0.05 was considered statistically significant.

## 3. Results

### 3.1. Patient Characteristics

**Figure 1:**
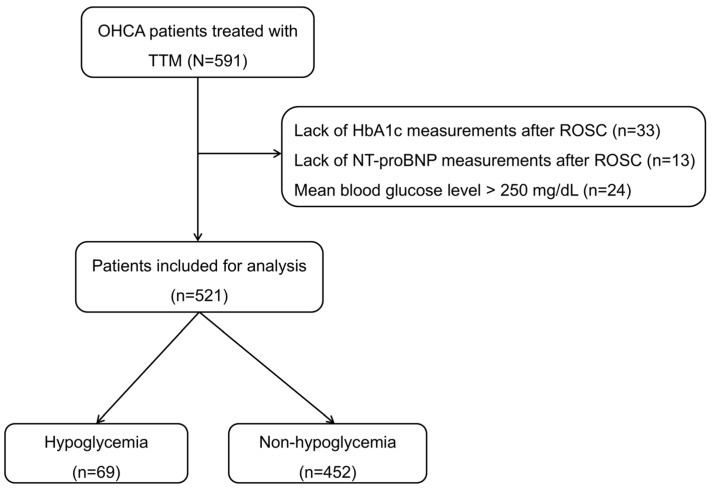
Flow diagram of patient inclusion. TTM, targeted temperature management; HbA1c, hemoglobin A1c; ROSC, restoration of spontaneous circulation; NT-proBNP, N-terminal pro-B-type natriuretic peptide.

A total of 591 OHCA survivors treated with TTM were identified. Of these, 521 met the inclusion criteria (Figure 1). The median age was 61.4 (50.1–70.7) years, and 69 patients (13.2%) experienced hypoglycemia.

Table 1 shows baseline characteristics stratified by hypoglycemia. Patients who experienced hypoglycemia had a lower BMI (22.0 vs. 23.5 kg/m^2^; *p* < 0.001); more hypertension (60.9% vs. 46.5%; *p* = 0.036) and diabetes (42.0% vs. 27.0%; *p* = 0.015); and fewer shockable rhythms (17.4% vs. 42.3%; *p* < 0.001) than those without hypoglycemia.

### 3.2. Comparison of Clinical Characteristics After ROSC According to the Occurrence of Hypoglycemia

**Figure 2:**
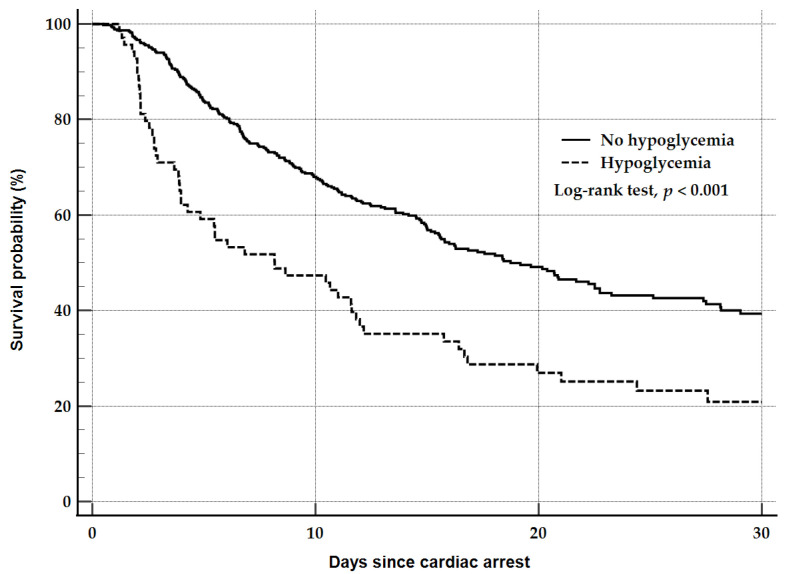
Kaplan–Meier survival curves after cardiac arrest in the hypoglycemia and no hypoglycemia groups.

After ROSC, patients with hypoglycemia had higher PaCO_2_ (54.0 vs. 42.0 mmHg; *p* < 0.001) and NT-proBNP (3088 vs. 189 pg/mL; *p* < 0.001) levels and more frequent RRT (55.1% vs. 17.5%; *p* < 0.001) than those without hypoglycemia (Table 2). The target temperature, seizure occurrence, and ECMO use did not differ between groups. Patients with hypoglycemia also had higher in-hospital mortality (79.7% vs. 53.5%; *p* < 0.001) and poorer neurological outcomes (85.5% vs. 68.8%; *p* = 0.007) than those without hypoglycemia. The survival curves of the hypoglycemia and no hypoglycemia groups were statistically different according to the log-rank test (*p* < 0.001) (Figure 2).

### 3.3. Analysis of Factors Associated with the Occurrence of Hypoglycemia

After adjusting for confounders, BMI (OR, 0.877; 95% CI, 0.808–0.953), NT-proBNP > 2000 pg/mL (OR, 4.155; 95% CI, 2.278–7.579), and RRT (OR, 3.741; 95% CI, 2.024–6.913) were independently associated with hypoglycemia (Table 3).

**Figure 3:**
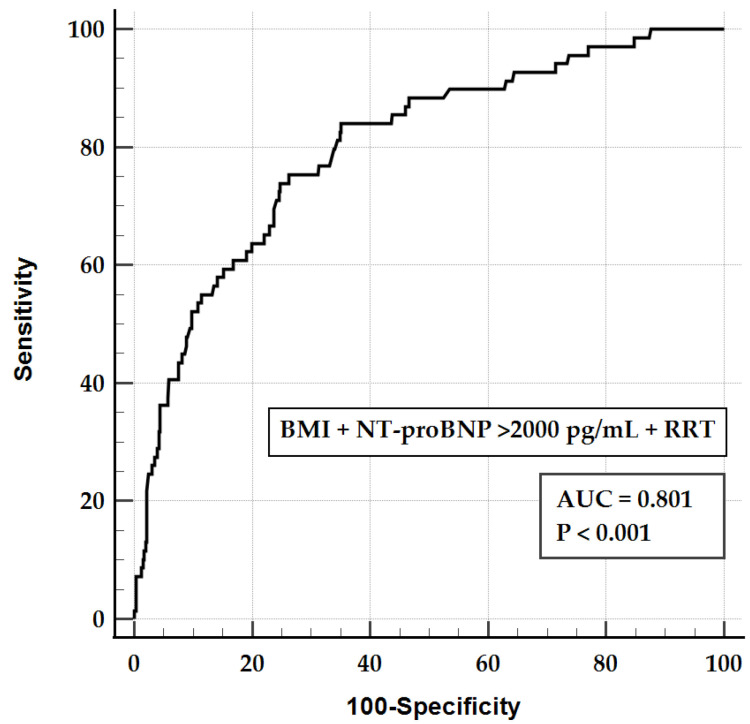
ROC curve of the final adjusted regression model predicting hypoglycemia. ROC, receiver operating characteristic; BMI, body mass index; NT-proBNP, N-terminal pro-B-type natriuretic peptide; RRT, renal replacement therapy; AUC, area under the curve.

Figure 3 depicts the AUROC for Model 1 when predicting hypoglycemia. The areas under the curves for BMI, NT-proBNP > 2000 pg/mL, RRT, and Model 1 were 0.637 (95% CI, 0.594–0.678), 0.710 (95% CI, 0.669–0.748), 0.688 (95% CI, 0.646–0.728), and 0.801 (95% CI, 0.764–0.835), respectively. The area under the curve for Model 1 was significantly higher than that for BMI, NT-proBNP > 2000 pg/mL, and RRT.

## 4. Discussion

Hypoglycemia occurred in 13.2% (69/521) of this cohort and was associated with a lower BMI, NT-proBNP > 2000 pg/mL, and RRT in the OHCA survivors treated with TTM. The final adjusted model demonstrated good discrimination (AUROC, 0.801; 95% CI, 0.764–0.835).

The BMI was independently associated with hypoglycemia in the present study. In intensive care unit populations, a higher BMI has been associated with lower hospital mortality and lower hypoglycemia rates [18]. Several mechanisms can explain this association. Insulin resistance in individuals with a higher BMI would contribute to maintaining relatively elevated blood glucose levels, providing a buffer against hypoglycemia [18]. Furthermore, adipose tissue can modulate inflammatory responses during critical illness [18]. In a recent retrospective analysis of critically ill patients, those with hypoglycemia had a significantly lower BMI (23.6 ± 4.6 kg/m^2^) than those without hypoglycemia (24.5 ± 4.7 kg/m^2^; *p* < 0.001) [19]. In a multivariate Cox regression, each 1 kg/m^2^ increase in the BMI was associated with a reduced mortality risk (hazard ratio, 0.980; 95% CI, 0.972–0.988; *p* < 0.001) [19]. Importantly, this protective effect was observed despite higher rates of hyperglycemia in patients with an elevated BMI [19], suggesting that the reduced hypoglycemia risk contributed to explaining the survival benefit associated with a higher BMI in critically ill populations. These findings demonstrate that the BMI is a key determinant of glucose control in critical illness. Given that OHCA survivors experience extreme metabolic stress characterized by systemic ischemia–reperfusion injury and post-resuscitation syndrome, BMI-specific glucose monitoring strategies warrant consideration in this population.

In this study, patients who received RRT had a higher risk of hypoglycemia. Among the OHCA survivors, the need for RRT often denotes severe shock, with pulmonary edema secondary to renal failure, severe metabolic acidosis, or multiorgan dysfunction [20]. In this context, endogenous glucose production, primarily hepatic and renal gluconeogenesis, would be impaired [21,22]. Severe shock promotes immune cell-driven glycolysis and increases peripheral glucose uptake, predisposing patients to hypoglycemia [23]. Procedure-related factors during hemodialysis can further lower plasma glucose, including reduced insulin clearance, glucose loss into dialysate, and diffusion into red blood cells [24]. These established mechanisms are consistent with the higher hypoglycemia rates observed in patients undergoing RRT.

In this study, elevated NT-proBNP levels were associated with hypoglycemia. While no association was observed between NT-proBNP and hypoglycemia in healthy adults, hypoxia has been linked to higher NT-proBNP levels [25]. In a cohort of 253 patients with type 2 diabetes and 230 matched controls, higher NT-proBNP was associated with left ventricular dysfunction in patients with diabetes [26]. Left ventricular dysfunction reduces the cardiac output and, thus, hepatic blood flow. Reduced perfusion depletes hepatic glycogen stores, limiting hepatic glucose release and increasing the hypoglycemia risk [27]. Specifically, NT-proBNP levels above 2000 pg/mL were associated with significant left ventricular dysfunction and advanced heart failure [16]. Therefore, although the post-ROSC rise in NT-proBNP may not directly cause hypoglycemia, hypoxemia and left ventricular dysfunction (reflected by an elevated NT-proBNP) can act as risk factors for hypoglycemia. In addition, NT-proBNP levels rose with extracellular fluid overload, renal dysfunction, severe shock, or systemic hypoperfusion, all of which predispose the patient to hypoglycemia [28,29,30].

In our study, there was no significant association between the pre-morbid HbA1c levels and the development of hypoglycemia in survivors after a cardiac arrest. Several studies in critically ill populations have reported a positive association, identifying high HbA1c as an independent risk factor for hypoglycemia [31,32]. In contrast, Bang et al. found no significant difference in the incidence of hypoglycemia between patient groups stratified by HbA1c levels in a cohort of post-cardiac arrest survivors [33]. Similarly, there was no significant association between HbA1c and severe hypoglycemia in a large-scale cohort study of adult type 2 diabetic patients [34]. These discrepant findings can be explained by differences in patient populations and clinical contexts. In the post-cardiac arrest patients who received TTM, the overwhelming impact of acute illness severity overshadowed the influence of chronic glycemic control. In the acute phase following ROSC, the profound metabolic derangements caused by multi-organ dysfunction, shock, and systemic inflammation would be more prevalent factors for hypoglycemia than the chronic glycemic state of patients [35,36]. Therefore, the predictive value of the HbA1c was masked by these acute-phase risk factors during post-cardiac arrest care.

This study has several limitations. First, as a single-center observational study, institutional variation in post-cardiac arrest management may limit the generalizability. Second, although we identified associations between the candidate risk factors and hypoglycemia, causality cannot be inferred because of the retrospective design. Third, blood glucose was measured hourly initially and every 4 h thereafter rather than continuously. This intermittent sampling may have missed transient hypoglycemic episodes between measurements, potentially underestimating the true incidence. Fourth, several factors may have influenced the observed associations between risk factors and hypoglycemia. BMI could be influenced by nutritional status, muscle mass distribution, and preexisting insulin resistance. NT-proBNP and RRT may be influenced by fluid status, the degree of acute kidney injury, vasopressors, and preexisting medications. Future prospective studies are needed to examine the relationship between these confounding factors and hypoglycemia. Finally, approximately 8% of patients were excluded because of missing NT-proBNP or HbA1c data, potentially introducing selection bias. Prospective studies incorporating continuous glucose monitoring and comprehensive metabolic assessment are warranted to validate our findings and establish optimal glucose management strategies for different risk groups.

## 5. Conclusions

In this study, BMI, NT-proBNP > 2000 pg/mL, and RRT were associated with hypoglycemia in patients with OHCA treated with TTM. These readily available clinical parameters demonstrated good predictive performance and can be used to identify patients at high risk of hypoglycemia during post-resuscitation care. These findings underscore the need to recognize and manage these risk factors, which may improve metabolic stability and potentially affect neurological and clinical outcomes. Future research should focus on developing and validating risk-adapted glucose management protocols, incorporating continuous glucose monitoring technology, and investigating the causal relationship between hypoglycemia prevention and long-term neurological outcomes in post-cardiac arrest patients.

## Figures and Tables

**Table 1:** Baseline characteristics by hypoglycemia status within 7 days.

Variables	Total (n = 521)	Hypoglycemia (n = 69)	No Hypoglycemia (n = 452)	*p*-Value
Demographics				
Age, years	61.4 (50.1–70.7)	63.3 (51.7–75.5)	61.2 (49.5–70.2)	0.094
Male, n (%)	378 (72.6)	51 (73.9)	327 (72.3)	0.899
BMI, kg/m^2^	23.2 (20.9–25.8)	22.0 (19.7–23.8)	23.5 (21.2–26.0)	<0.001
Preexisting conditions, n (%)				
Coronary artery disease	74 (14.2)	12 (17.4)	62 (13.7)	0.529
Prior stroke or TIA	44 (8.4)	5 (7.2)	39 (8.6)	0.879
Hypertension	252 (48.4)	42 (60.9)	210 (46.5)	0.036
Diabetes	151 (29.0)	29 (42.0)	122 (27.0)	0.015
Chronic pulmonary disease	42 (8.1)	6 (8.7)	36 (8.0)	1.000
Malignancy	31 (6.0)	4 (5.8)	27 (6.0)	1.000
Cardiac arrest characteristics				
Witnessed collapse, n (%)	331 (63.5)	43 (62.3)	288 (63.7)	0.928
Bystander CPR, n (%)	323 (62.0)	48 (69.6)	275 (60.8)	0.209
Shockable rhythm, n (%)	203 (39.0)	12 (17.4)	191 (42.3)	<0.001
Cardiac etiology, n (%)	316 (60.7)	36 (52.2)	280 (61.9)	0.157
Time to ROSC, min	28.0 (18.0–43.0)	33.0 (18.5–45.0)	28.0 (18.0–42.0)	0.541

BMI, body mass index; CPR, cardiopulmonary resuscitation; ROSC, return of spontaneous circulation; TIA, transient ischemic attack.

**Table 2:** Clinical characteristics after ROSC by hypoglycemia status within 7 days.

Variables	Total (n = 521)	Hypoglycemia (n = 69)	No Hypoglycemia (n = 452)	*p*-Value
Immediate post-ROSC period				
Lactate, mmol/L	8.3 (5.2–11.8)	9.4 (6.0–12.7)	8.0 (5.1–11.6)	0.084
Glucose, mg/dL	254 (189–324)	222 (172–320)	263 (194–332)	0.115
HbA1c, %	5.7 (5.4–6.2)	5.8 (5.4–6.8)	5.7 (5.3–6.1)	0.088
PaO_2_, mmHg	145.0 (89.0–236.0)	138.0 (83.4–225.5)	148.0 (90.3–236.8)	0.216
PaCO_2_, mmHg	43.4 (34.3–58.5)	54.0 (38.5–66.5)	42.0 (34.0–56.0)	<0.001
NT-proBNP level, pg/mL	237 (64–1651)	3088 (208–10,000)	189 (56–1059)	<0.001
NT-proBNP > 2000 pg/mL, n (%)	120 (23.0)	41 (59.4)	79 (17.5)	<0.001
Hyperglycemia within 7 days, n (%)	269 (51.6)	38 (55.1)	231 (51.1)	0.628
TTM target temperature, n (%)				0.464
33.0 °C	474 (91.0)	61 (88.4)	413 (91.4)	
34.0–35.0 °C	12 (2.3)	3 (4.3)	9 (2.0)	
36.0 °C	35 (6.7)	5 (7.2)	30 (6.6)	
Seizure, n (%)	145 (27.8)	18 (26.1)	127 (28.1)	0.839
ECMO, n (%)	41 (7.9)	5 (7.2)	36 (8.0)	1.000
RRT, n (%)	117 (22.5)	38 (55.1)	79 (17.5)	<0.001
In-hospital mortality, n (%)	297 (57.0)	55 (79.7)	242 (53.5)	<0.001
Poor neurological outcome, n (%)	370 (71.0)	59 (85.5)	311 (68.8)	0.007

ROSC, restoration of spontaneous circulation; HbA1c, hemoglobin A1c; PaO_2_, partial pressure of oxygen; PaCO_2_, partial pressure of carbon dioxide; NT-proBNP, N-terminal pro-B-type natriuretic peptide; TTM, targeted temperature management; ECMO, extracorporeal membrane oxygenation; RRT, renal replacement therapy.

**Table 3:** Multivariable logistic regression for hypoglycemia within 7 days.

Variables	OR (95% CI)	*p*-Value
BMI	0.877 (0.808–0.953)	0.002
Hypertension	1.091 (0.569–2.093)	0.792
Diabetes	0.806 (0.419–1.552)	0.519
Shockable rhythm	0.500 (0.249–1.003)	0.051
PaCO_2_, mmHg	1.011 (0.997–1.025)	0.131
NT-proBNP > 2000 pg/mL	3.769 (2.060–6.898)	<0.001
RRT	3.429 (1.841–6.387)	<0.001

OR, odds ratio; CI, confidence interval; BMI, body mass index; PaCO_2_, partial pressure of carbon dioxide; NT-proBNP, N-terminal pro-B-type natriuretic peptide; RRT, renal replacement therapy.

## Data Availability

The data presented in this study are available upon request from the corresponding author. The data are not publicly available due to personal protection.

## Abbreviations

The following abbreviations are used in this manuscript:

OHCA	Out-of-hospital cardiac arrest
ROSC	Return of spontaneous circulation
TTM	Targeted temperature management
HbA1c	Hemoglobin A1c
NT-proBNP	N-terminal pro-B-type natriuretic peptide
BMI	Body mass index
CPR	Cardiopulmonary resuscitation
PaCO_2_	Partial pressure of carbon dioxide
ECMO	Extracorporeal membrane oxygenation
RRT	Renal replacement therapy
CPC	Cerebral Performance Category
OR	Odds ratio
CI	Confidence interval
AUROC	Area under the receiver operating characteristic curve
TIA	Transient ischemic attack
AUC	Area under the curve
